# Introduction to the special section on predictors and outcomes of peer victimization

**DOI:** 10.1177/01650254251361351

**Published:** 2025-08-07

**Authors:** Claire F. Garandeau, Sarah T. Malamut, Lydia Laninga-Wijnen

**Affiliations:** 1 University of Turku, Finland

**Keywords:** Peer victimization, internalizing, genetic confounding, parenting, school environment, academic self-efficacy, sleep problems, peer status, implicit attitudes

## Abstract

The eight longitudinal studies published in this special section rely on data from five countries and on rigorous methodologies (such as genetically sensitive, multilevel, or experimental designs) to shed light on predictors and outcomes of peer victimization at school. This introduction highlights the main questions addressed by the current set of studies. First, we present studies which sought to identify the factors that a) put children and youth at higher risk of victimization by peers and b) exacerbate the effects of these vulnerability factors, as well as to determine whether genes account for some of these effects. Second, we present studies focusing on the links between peer victimization and later biological, psychological and social adjustment and on the possible moderators and mediators of these associations. Together, these studies help us better understand the maladaptive cycle of peer victimization, which can inform the development of school-based and targeted interventions.

Peer victimization is the experience of being repeatedly the target of aggressive behaviors by one or more peers. There is ample evidence that being victimized by peers in childhood and adolescence is negatively associated with future mental well-being (Christina et al., 2021), academic achievement (e.g., Liu et al., 2014), physical health (Schacter, 2021) and social adjustment (Demol et al., 2020). Moreover, some of these adverse outcomes put children and youth at risk for peer victimization (Christina et al., 2021; Demol et al., 2020), suggesting a self-reinforcing maladaptive cycle. In order to break this detrimental cascade effect, more knowledge is needed (1) on the mechanisms explaining why peer victimization might lead to these outcomes as well as why some of these negative characteristics can increase the likelihood of being victimized, and (2) on the personal and contextual factors that can mitigate or exacerbate these bidirectional associations between maladjustment and peer victimization.

Thus, the aim of this special section is to enhance our understanding of the mediators and moderators of the effects of peer victimization on a wide range of psychological, social, or physical outcomes as well as the effects of vulnerability factors on peer victimization. To achieve this goal, all eight studies included in this section tested prospective associations. This set of studies is also characterized by its use of rigorous methodologies, including genetically sensitive research designs that control for genetic factors, multilevel designs that examine interactions between personal and contextual factors, long-term longitudinal designs to test the effects of adolescent victimization into adulthood, as well as experimental designs to capture implicit/automatic processes. The data analyzed across the studies were collected in various countries, including Canada, Finland, Germany, the Netherlands, and the United States.

## Who Is More at Risk for Peer Victimization?

The studies in this special section provide further elucidation of factors that can make children and youth more vulnerable to peer victimization, specifically: internalizing symptoms and low peer status (Garandeau et al., 2025), being a newcomer in the school (Tenhunen et al., 2025) and exposure to negative parenting (Oncioiu et al., 2025). Three important questions were addressed in relation to these risk factors.

### Does the Context Matter?

Differential susceptibility theory (Belsky & Pluess, 2009) suggests that the extent to which psychosocial factors pose a risk for victimization depends on the context. Two studies in this special section investigated the role of classroom and school contexts and suggest that the social environment can make a difference for vulnerable students. Using multilevel analyses and data collected at the beginning and in the middle of the school year among Finnish adolescents, Garandeau et al. (2025) examined whether students suffering from psychosocial difficulties (internalizing problems and peer rejection) were more at risk in some classrooms than in others. The concurrent and prospective effects of internalizing symptoms and peer rejection on both self- and peer-reported peer victimization were found to vary significantly across classrooms. Although it was hypothesized that the effects of these vulnerability factors on victimization would be exacerbated in classrooms where these risk factors are less common or in smaller classrooms (where these risk factors may stand out more), none of these classroom characteristics explained this between-classroom variation. These findings highlight that the classroom context might play an important role in determining whether well-known vulnerability factors are related to future victimization, and it is critical for future research to identify the contextual characteristics driving or buffering against this effect.

When a student is victimized by peers, it is common for them and those who care about them to wonder whether a change of school would put an end to their plight or place them in an even more vulnerable position. Using an exceptionally large sample of more than 58,000 children and adolescents attending Finnish schools, Tenhunen et al. (2025) shed light on this question by investigating whether being a newcomer in the school put students even more at risk when they had a history of being victimized in their previous environment. Results from quantitative analyses showed that newcomer status was positively associated with current self-reported victimization but also showed that this association was moderated by victimization history. The newcomers who had been constantly or frequently victimized in their previous school were significantly less victimized than established students with similar victimization histories. This suggests that a school change may be beneficial for chronically victimized students. Interviews with a subsample of 68 students provided a slightly more nuanced view by revealing that some of the newcomers who had been victimized in their former school did not benefit from the change, especially if they had lost friends and were unable to make new ones. The change also appeared to be more beneficial to those who were frequent victims than to those who were victimized occasionally. Further research comparing youth with comparable victimization history who switched schools to those who remained in the same school would be helpful to reveal whether previously victimized newcomers experience more or less victimization than they would have faced in their previous schools.

### Do Genes Matter?

Several studies in the literature found support for an association between exposure to negative parenting and being victimized by peers (see Nocentini et al., 2019). However, these associations may be confounded by genetic factors as parents provide both genes and environment to their children and most studies did not examine this possibility (Kretschmer, 2023). The study by Oncioiu et al. (2025) addressed this limitation by investigating whether the effect of exposure to negative parenting on peer victimization was explained by genetic and shared environmental factors, using a sample of same-sex twin pairs from a longitudinal population-based study conducted in Germany, including a 5-year-old and an 11-year-old cohort. When considering all participants in the sample, this study found significant associations between exposure to negative parenting behavior and a higher chance of being the target of peer aggression. To determine whether this association was confounded by genetics and shared environmental factors, this study employed a co-twin control design that allows testing whether monozygotic twins who differ in exposure to negative parenting behavior differ in peer victimization. Specifically, this study was interested in the “within-twin pair” effect, which represents the variation in peer victimization accounted for by the level of negative parenting behavior of one twin relative to their co-twin. Indeed, within-twin pair differences in negative parenting being significantly associated with within-twin pair differences in peer victimization, would suggest a causal effect of parenting behavior. However, after accounting for genetic and shared environmental factors in the analyses with monozygotic twins, the association between exposure to negative parenting and peer victimization became non-significant. This indicates that the findings of prior studies (e.g., Lereya et al., 2013) showing links between negative parenting behavior and bullying victimization may have been inflated due to not accounting for the confounding of genetic factors.

### Do Age and Sex Matter?

The effects described above may differ for children and adolescents. Tenhunen et al. (2025) did pay attention to the potential role of age in the effects of school change. Among students who had been constantly victimized, changing schools was more beneficial for elementary-school than for middle-school students. Those who had been victimized *almost* constantly benefited from a school change only at the elementary-school level. When the victimization was less frequent, the school change was actually associated with a higher risk for victimization at the middle-school level. In line with findings that adolescents are more resistant than children to anti-bullying interventions (Yeager et al., 2015), changing schools also seems to be a less effective strategy in adolescence. Regarding the influence of parenting, it is often assumed to decrease in adolescence when peers become increasingly important. However, surprisingly, no evidence was found by Oncioiu et al. (2025) that the effects of negative parenting on peer victimization were lower for adolescents than for children.

As boys and girls might respond differently to parenting, Oncioiu et al. (2025) tested for sex differences in the association of negative parenting behavior with peer victimization and found that, in early adolescence, negative parenting behavior remained associated with peer victimization among monozygotic twin girls but not among monozygotic twin boys. However, this finding was based on a small sample, so further research is needed to confirm these sex differences.

## How and for Whom Does Peer Victimization Predict Future Adjustment?

### Which Outcomes Did Peer Victimization Predict?

Among the studies focusing on the prospective effects of peer victimization, some evidence was found that peer victimization was linked to detrimental effects for the outcomes and the time intervals under investigation. Self-reported peer victimization across ninth grade was associated with several indicators of sleep difficulties at the beginning of Grade 10, as well as lower academic efficacy (i.e., lower confidence in their academic abilities) in the middle of Grade 10 in a US sample (Bakth et al., 2025). A different study, however, found that a trajectory of high (self-reported) victimization from age 12 to 17 was not, in itself, linked to abnormal levels of hair cortisol concentration (HCC) at age 19 in a Quebec sample (Brendgen et al., 2025). In that study using a genetically informed twin design, differences in the outcome (HCC) were mostly explained by genetic factors and shared environmental factors. In the study by Lansu et al. (2025) conducted with a Dutch sample of third to sixth graders, being a victim of peer bullying was associated with more reactive aggression a few months later, but had no significant longitudinal association with sadness, social withdrawal, or anger. Finally, peer victimization at age 11 (regardless of informant) did not predict lower self-esteem or higher internalizing problems at age 19 in a Dutch sample (Wiertsema et al., 2025). The effects of peer victimization on later parenting depended on the informant for victimization and the indicator of parenting. Specifically, self-reported victimization in early adolescence predicted less parental self-efficacy while peer-reported victimization -unexpectedly- predicted less parental stress.

### Moderating Factors

Three studies provided insights on possible moderators of the prospective effects of peer victimization, with two studies focusing on the influence of peers (Brendgen et al., 2025; Lansu et al., 2025) and one on the influence of teacher characteristics (Brigham et al., 2025). First, the study by Brendgen et al. (2025) highlighted the important role of social support in a sample of monozygotic and dizygotic twin pairs. Combining Growth Mixture and Biometric modeling, they showed that those in the stable high victimization trajectory did experience a blunted pattern of cortisol secretion when they received little help from friends, but not when they enjoyed high levels of friendship support. As cortisol hyposecretion is linked with a wide range of mental and physical health problems (Brindle et al., 2022), these findings show that supportive friendships could protect chronic victims from a multitude of negative outcomes. Support from parents, however, did not show any protective effect, thus the study of Brendgen et al. (2025) suggests the need for youth exposed to harmful peer behavior to find support within the peer world. However, prior literature has yielded inconsistent findings regarding the beneficial effects of supportive friends for victimized youth (see Schacter et al., 2021 for a review); thus, further research should seek to determine under which conditions and for which outcomes these friendships help or hurt.

Second, using mixed-effect modeling, the study by Lansu et al. (2025) focused on whether peers’ implicit and explicit attitudes and behaviors might be related to worse psychological and social outcomes among victims and showed that peer-victimized children displayed more reactive aggression a few months later when they had been exposed to more uncivil deliberate behavior intentions from classmates. However, these negative behavioral intentions were not associated with any other well-being indicators, neither were the other attitudes and behaviors under investigation, whether implicit or explicit.

Third, as teachers are expected to play a significant role in prevention and handling of bullying and their interventions are generally helpful (e.g., Burger et al., 2022), the study by Brigham et al. (2025) focused on children’s disclosure of their victimization to teachers. Indeed, many victimized youth do not talk to their teacher (Blomqvist et al., 2020) and it is important to understand why. The study by Brigham et al. (2025) sought to identify, in a sample of US fourth and fifth graders, the factors that predict whether children seek assistance from the teacher when peers are mean to them. First, latent change modeling analyses were used to detect within-person changes in assistance-seeking across one school year. Among all participants, assistance-seeking tended to decrease during the year, but less so for those who reported being highly victimized. Second, the way teachers generally handle cases of bullying as well as the quality of their relationship with their students turned out to be significantly related with assistance-seeking. Children were more likely to seek assistance when they perceived their teacher as using the following strategies to handle bullying situations: involving parents, punishing aggressors, separating students, advising avoidance, and advising assertion. However, differences emerged depending on students’ gender and type of victimization. At the beginning of the school year, girls reported greater assistance-seeking and decreased less than boys in assistance-seeking over the year. Overtly victimized children were more likely to seek assistance throughout the year when they were close to their teacher but decreased in assistance-seeking when their closeness to the teacher was low. Overtly victimized boys reported more assistance-seeking across the school year when their relationship with the teacher was not conflictual and decreased in assistance-seeking if this relationship was highly conflictual. Surprisingly, highly relationally victimized girls reported more assistance-seeking across the school year if they had low—compared with high—levels of teacher–child closeness. Taken together, the findings suggest that children’s perceptions of how their teacher typically responds to peer victimization matters more for their assistance-seeking than how close or conflictual their relationship is with their teacher.

### Mediating Factors

Regarding possible pathways explaining why victimization may be harmful, the study by Bakth et al. (2025) shed light on the role of sleep in the links between peer victimization and later academic adjustment in adolescence. Specifically, findings indicated that adolescents experiencing greater peer victimization during ninth grade experienced increased sleepiness and lethargy interfering with day-to-day activities (i.e., daytime dysfunction) as well as increased sleep disturbances (e.g., having nightmares) at the beginning of 10th grade, which, in turn, predicted poorer academic efficacy in the middle of 10th grade.

While Bakth et al. (2025) investigated mechanisms explaining the associations of victimization with future adjustment within a relatively short timeframe, Wiertsema et al. (2025) focused on links between peer victimization and outcomes in the long term by testing whether individuals’ experiences of peer victimization in early adolescence predicted their own parenting as adults and whether this could be explained by the links between early-adolescence victimization and late-adolescence self-esteem and internalizing problems. However, the results did not support such associations. This could be due to the fact that the measure of victimization in that study did not sufficiently capture its frequency and persistency. Moreover, as these variables were measured over two decades, this could indicate that some of the detrimental effects of peer victimization, which are well-documented in the short term, may not be as present after longer periods of time.

## Conclusions

This special section provided important replications of prior findings concerning possible risk factors and prospective effects of victimization by peers. However, some of the expected associations were not supported and we believe this special section highlights several factors that may explain this. A key strength of the studies presented here is their longitudinal designs. Although the link between victimization and adjustment is well-studied, a vast majority of this research focuses on concurrent associations, especially those studies considering moderating factors. Thus, this special section sheds light on the temporal precedence of maladjustment and victimization.

Across three studies on predictors of peer victimization, evidence was found that psychological factors (internalizing problems; history of peer victimization) and social factors (peer rejection, school transitions, and negative parenting) predicted victimization at a later time point while controlling for prior victimization. However, the association of negative parenting with later victimization became non-significant after controlling for genetic factors. Future work is encouraged to identify more characteristics that make students vulnerable to victimization after accounting for important covariates. In addition, future research should further investigate which classroom features explain why vulnerable adolescents are more at risk in some classrooms than in others, as well as consider variability beyond the classroom level, by also examining the role of norms in friendships or cliques.

Five of the studies focused on the longitudinal effects of victimization on future social, psychological, and physical adjustment while controlling for these outcomes at earlier time points. Although victimization was related to some adverse outcomes, not all expected associations were supported, and effect sizes were overall smaller than what is typically reported in the literature. The use of stringent methodologies, such as genetically sensitive designs, may have contributed to this. In fact, multiple studies in this special section highlight the importance of considering potential genetic confounding—that is, that the same genes could explain both the predictor and outcome. This is not to say that the possible detrimental effects of victimization are only present due to genetic confounding, but accounting for this possibility can provide a more representative estimate of the associations between victimization and adverse outcomes. Moreover, the studies mostly used recently collected data that must adhere to new ethical standards, such as relying on active parental consent procedures, which could lead to biased samples (e.g., under-representation of students with problem behaviors) and biased associations between outcomes of interest. Indeed, the use of active (versus passive) consent was found to moderate links between victimization and social-emotional variables (Shaw et al., 2015). Furthermore, including measures capturing the persistence of victimization might be key in predicting longer-term outcomes (McDougall & Vaillancourt, 2015). For instance, students victimized at school may have a higher chance of being harassed in the workplace later in life, which might contribute to observed prospective links between peer victimization and future long-term adjustment. Moreover, to better understand the shorter-term mechanisms underlying these longitudinal associations, more research should also consider particularly short timeframes via the use of daily diary studies (e.g., Laninga-Wijnen et al., 2024) or ecological momentary assessments (EMAs).

Through their advanced methodologies and their use of large samples and recently collected data from various countries, the present set of studies contributes to our understanding of how personal and contextual characteristics expose some youth to harassment from their peers, the specific outcomes it might have on their physical, psychosocial adjustment and coping mechanisms, and sheds light on some of the underlying mechanisms explaining these effects. We hope that it will inspire further rigorous research on the topic and ultimately inform the design of anti-bullying interventions and policies.
